# The CDH1 -160C/A polymorphism is associated with breast cancer: evidence from a meta-analysis

**DOI:** 10.1186/s12957-016-0927-0

**Published:** 2016-06-27

**Authors:** Ying-Yu Ma, Wei-Quan Wu, Zheng-Chuang Liu, Xiao-Fen Yu, Kun Guo, Qi-Wen He, Shi-Bin Jiang, Qin-Shu Shao, Hou-Quan Tao, Dong-Sheng Huang

**Affiliations:** Key Laboratory of Gastroenterology of Zhejiang Province, Zhejiang Provincial People’s Hospital, Hangzhou, 310014 China; Digestive System Department, Zhejiang Provincial People’s Hospital, Hangzhou, 310014 China; Operation Room, Zhejiang Provincial People’s Hospital, Hangzhou, 310014 China; Wenzhou Medical University, Wenzhou, 310025 Zhejiang Province China; Department of Gastrointestinal Surgery, Zhejiang Provincial People’s Hospital, Hangzhou, 310014 China

**Keywords:** CDH1, Polymorphism, Breast cancer, Meta-analysis

## Abstract

**Background:**

Numerous epidemiological studies have evaluated the association between the CDH1 -160C/A polymorphism and the risk of breast cancers. However, these studies have yielded conflicting results. To derive a more precise estimation of this association, this meta-analysis was conducted.

**Methods:**

A comprehensive search using the keywords “CDH1,” “E-Cadherin,” “polymorphism,” “SNP,” and “variant” combined with “breast,” “cancer,” “tumor,” or “carcinomas” was conducted. Pooled odds ratios (ORs) with 95 % confidence intervals (CIs) were appropriately calculated using a fixed effect or random effect model. Preferred Reporting Items for Systematic Reviews and Meta-Analyses (PRISMA) 2009 checklist was used for this meta-analysis.

**Results:**

Four publications including five studies were identified. It was found that the CDH1 -160C/A polymorphism was significantly associated with breast cancer risk in the dominant model (CA + AA vs. CC: OR = 1.207, 95 % CI = 1.031–1.412, *P* = 0.019).

**Conclusions:**

Our meta-analysis demonstrated that the -160C/A polymorphism in the CDH1 gene might contribute to breast cancer susceptibility. Further investigations using a much larger sample including different ethnicities are still needed to verify this association.

**Electronic supplementary material:**

The online version of this article (doi:10.1186/s12957-016-0927-0) contains supplementary material, which is available to authorized users.

## Background

E-cadherin (*CDH1*), located on chromosome 16q22.1, is one of the most important tumor suppressor genes encoding an adhesion glycoprotein [1, 2]. Studies showed that CDH1 plays a critical role in intercellular adhesion, cell polarity, cell signaling, and maintenance of normal tissue morphology and cellular differentiation, which is expressed in almost all epithelial cells [3–5]. E-cadherin expression loss has been described in gynecological neoplasias, and *CDH1* silencing by promoter methylation has been detected in both cervical and endometrial tumors [6, 7].

Several polymorphisms have been identified within the *CDH1* gene, and emerging numbers of studies show that genetic variations within the E-cadherin gene are involved in oncogenesis and development [8–11]. The -160C/A (rs16260) is an important functional SNP located in the promoter region that has significant effects on transcriptional activity in transient transfection studies [8–10]. Previous studies have indicated the association of the -160C/A polymorphism with the risk and progression of various human cancers [4]. However, the polymorphism’s genetic effects vary in different cancer types. Recent epidemiologic studies revealed an increased cancer risk for the A (-160) allele carriers [12–15] and a protective role in gastric cancer [16], while the others showed no significant association [17–21]. Researchers report inconsistent breast cancer results; some studies found no association among -160A allele carriers [17, 22], while others found a significantly increased risk [23]. Cattaneo et al. found A allele carriers significantly increased risk for endometrial cancer [24] but not for cervical and ovarian cancer [24, 25]. Thus, these observations raised a controversial question regarding the significance of -160C/A in cancer pathogenesis, especially in breast cancer. Obviously, an individual study’s statistical power could be very limited for efficient assessment of the -160A allele. Integration of these data sets may provide improved statistical power to detect the significance.

## Methods

### Publication selection

Studies examining the association between the CDH1 -160C/A polymorphism and breast cancer were systematically searched using the following key words: “CDH1,” “E-Cadherin,” “polymorphism,” “SNP,” and “variant” combined with “breast,” “cancer,” “tumor,” or “carcinomas,” and the studies were identified via an electronic search on PubMed, Elsevier, Springer, Wiley, EBSCO, OVID, and Google scholar. Publication titles and abstracts were carefully reviewed. We also manually searched and verified the references of those retrieved articles to obtain other relevant publications. The last search included results up to July 2014.

### Inclusion and exclusion criteria

Studies for further meta-analysis were selected based on the following criteria: (1) original epidemiological studies on the association between the CDH1 promoter polymorphsim and breast cancer patients; (2) case-control studies; (3) studies containing at least two comparison groups (cancer group vs. control group); or (4) detailed genotyping data. For duplicated studies, only those with the largest sample size in our present study were included.

The following criteria to exclude studies were used: (1) studies from which genotype frequencies or alleles could not be obtained; (2) studies on family members; (3) systematic reviews or abstracts; (4) animal studies; (5) non-English language; and (6) <50 cases.

### Data extraction

Data were extracted from all eligible studies by two independent reviewers. Discrepancies were resolved by consulting with a third reviewer or the authors’ discussion. For each eligible study, publication details were collected based on the following aspects: name of the first author, year of publication, country of origin, cancer type, sources of control and case groups, genotyping methods for SNP, and the frequency of the genotypes and alleles of the CDH1 -160C/A polymorphism in cases and controls. For studies including subjects of different ethnic groups, data were extracted separately for each ethnic group whenever possible. Finally, PRISMA 2009 checklist (Preferred Reporting Items for Systematic Reviews and Meta-Analyses: The PRISMA Statement. PLoS Med 6(6): e1000097. doi:10.1371/journal.pmed1000097) was used for this meta-analysis.

### Statistical analysis

The wild type CC genotype of CDH1 -160C/A SNP was considered as a reference. Pooled effects were calculated for the codominant model (AA vs. CC; CA vs. CC), dominant model (CA + AA vs. CC), and recessive model (AA vs. CC + CA). The Hardy-Weinberg equilibrium (HWE) for each study’s control group was assessed using the goodness-of-fit test (*χ*^2^ or Fisher’s exact test).

The heterogeneity among included studies was evaluated using the Cochran’s Q-test and Higgins’ *I*^2^ statistic. Heterogeneity was defined as low when *P* ≥ 0.1, according to the Cochran’s Q-test, or when *I*^2^ was less than 50 %, according to the Higgins’ *I*^2^ statistic. A fixed effect model was then applied using the Mantel-Haenszel method. Otherwise, we applied a random effect model using the DerSimonian and Laird method. A pooled odds ratio (OR) with 95 % CI was used to assess the strength of association between the CDH1 -160C/A SNP polymorphism and cancer risk, depending on the heterogeneity of the analysis. A sensitivity analysis was performed to evaluate how robust the pooled effect size was at removing the effects of individual studies. An individual study was suspected to have excessive influence if the point estimate was outside the 95 % CI of the combined effect size after it was removed from the analysis. The potential publication bias among the included studies was assessed using the Egger test and Begg test. Statistical analysis was performed using STATA 11.0 software (Stata Corporation, College Station, TX, USA). All *P* values were two-sided, with *P* < 0.05 considered statistically significant.

## Results

### Literature screening and study selection

**Fig. 1:**
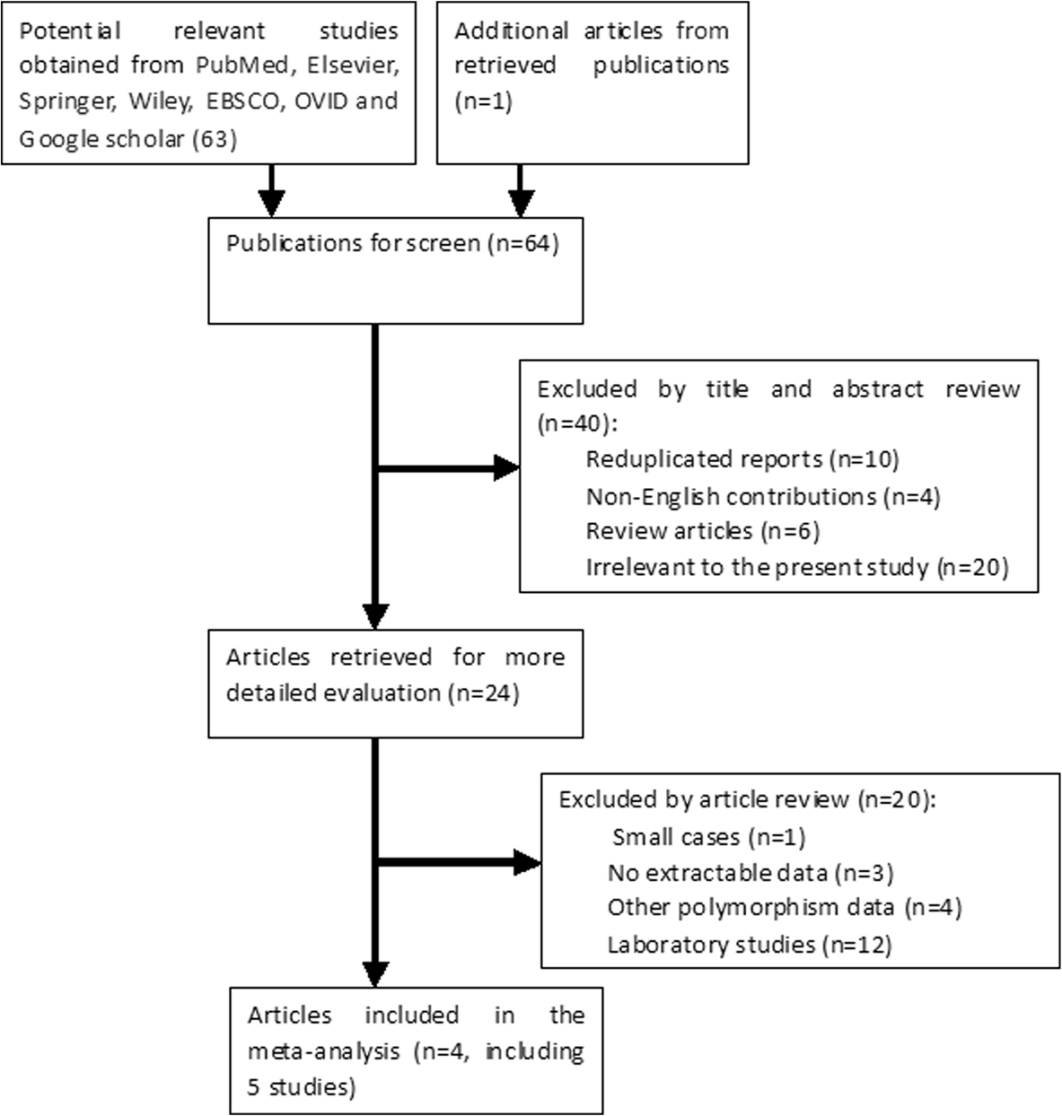
Flow chart of literature search and selection according to inclusion and exclusion criteria

After a preliminary on-line search, 64 potentially relevant articles were identified for further detailed evaluation. Twenty-four articles were selected for full-text review after 40 were excluded by manually screening the titles, abstracts, and key words for review articles, laboratory studies, reduplicated reports, non-English contributions, or those irrelevant to the current analysis. Finally, there were only four papers [17, 23, 24, 26] including five studies (1344 cases and 1569 controls involved) in the pooled analyses (Fig. 1) after 20 studies were excluded because they contained no extractable data, other polymorphism data, or review. Additionally, a list of excluded studies and reasons for exclusions was also provided in Additional file 1.

### Study characteristics

The main characteristics and basic information of eligible studies are summarized in Table 1. The studies enrolled 1344 patients from Switzerland, the Czech Republic, Italy, China, and India. Multiple genotyping methods were performed in the studies, including PCR-RFLP, PCR-SSCP, TaqMan PCR, and DNA sequencing. The distribution of genotypes in the controls of all studies was consistent with Hardy-Weinberg equilibrium, except in one study [26]. Then, the association between CDH1 -160C/A SNP and breast cancer risk was analyzed.

**Table 1:** CDH1 -160C/A SNP genotype distribution in cases and controls

Author-year	Country	Cancer type	Method	Genotype (N)						*P* HWE controls
				Case			Control			
				CC	CA	AA	CC	CA	AA	
Lei et al. 2002	Swiss	Breast	PCR-SSCP	226	166	32	135	92	21	0.350
Lei et al. 2002	Czech	Breast	PCR-SSCP	74	60	18	51	42	7	0.677
Cattaneo et al. 2006	Italy	Breast	PCR-RFLP	50	43	6	139	89	18	0.476
Yu et al. 2006	China	Breast	Taqman	222	201	44	243	187	39	0.721
Tipirisetti et al. 2013	India	Breast	Sequencing	120	26	56	175	42	33	<0.001

*PCR-SSCP* Polymerase chain reaction single-strand conformation polymorphism, *RFLP* restriction fragment length polymorphism

### Heterogeneity tests

The genotype data for breast cancer in the two studies were homogenous for the codominant genetic model (CA vs. CC: Q-test = 1.55, *P* = 0.818, *I*^2^ = 0.0 %) and the dominant genetic model (CA + AA vs. CC: Q-test = 2.95, *P* = 0.566, *I*^2^ = 0.0 %) (Table 2), and the fixed-effects model in these studies was employed. However, heterogeneity was significant for the codominant genetic model (AA vs. CC: Q-test = 8.41, *P* = 0.078, *I*^2^ = 52.5 %) and the recessive genetic model (AA vs. CC + CA: Q-test = 10.54, *P* = 0.032, *I*^2^ = 62.0 %) and allele comparison (A vs. C: Q-test = 11.20, *P* = 0.024, *I*^2^ = 64.3 %), and a random-effects model was performed.

**Table 2:** Meta-analysis of the association between CDH1 -160C/A polymorphism and breast cancer risk

Comparisons	Odds ratio	95 % confidence interval	*P* value	Heterogeneity		Effects model
				*I* ^2^ (%)	*P* value	
A vs C	1.231	0.992–1.528	0.060	64.3	0.024	Random
AA vs CC	1.397	0.922–2.116	0.115	52.5	0.078	Random
CA vs CC	1.116	0.941–1.325	0.208	0.0	0.818	Fixed
CA + AA vs CC	1.207	1.031–1.412	0.019	0.0	0.566	Fixed
AA vs CC + CA	1.338	0.850–2.105	0.208	62.0	0.032	Random

### Quantitative data synthesis

**Fig. 2:**
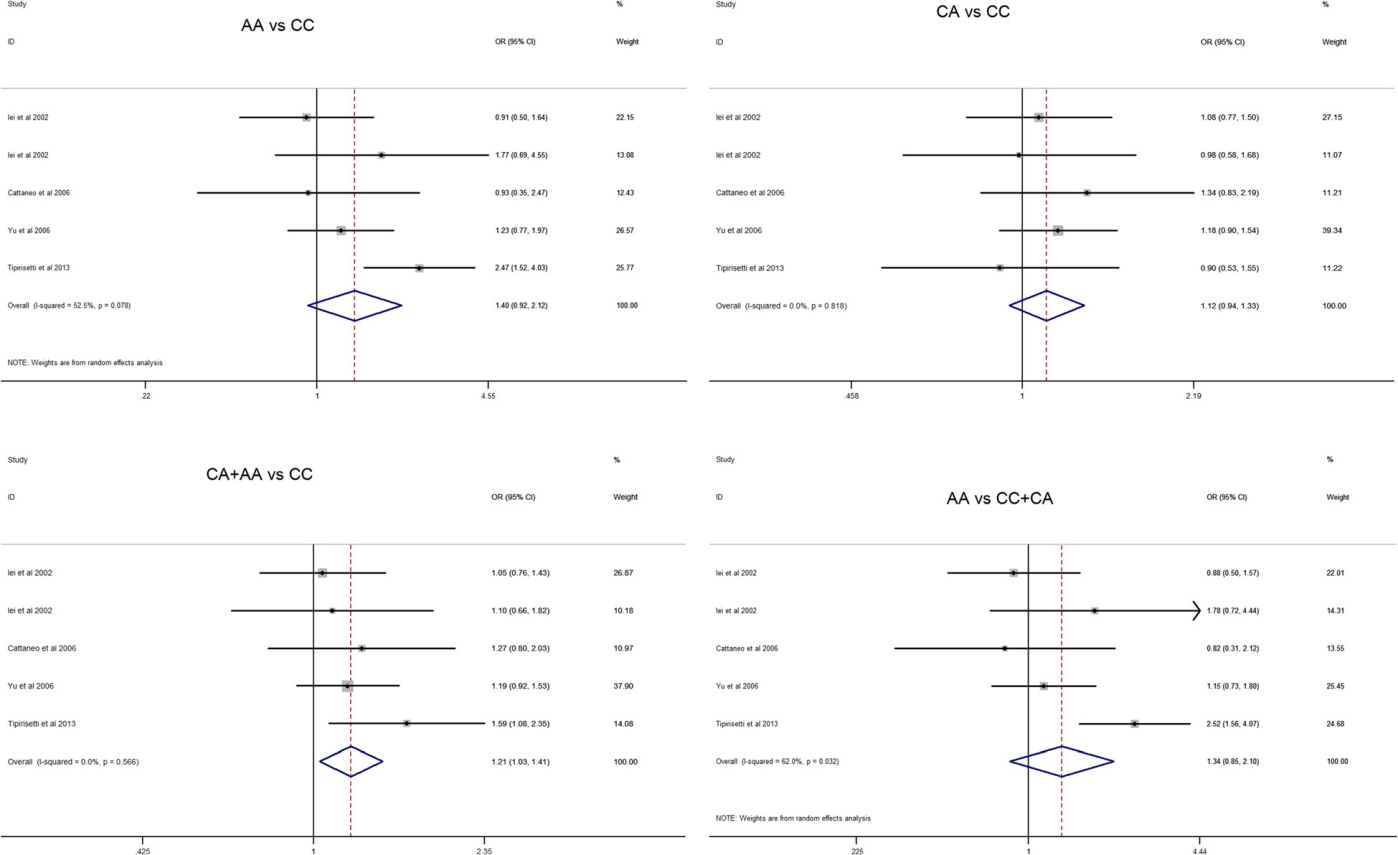
Forest plots of effect estimates for CDH1 -160 C/A polymorphism in different genetic models. For each of the studies, the *boxes* and *horizontal lines* represent the OR and the corresponding 95 % CI; the *area of the boxes* indicates the weight (inverse of the variance). The *diamond* corresponds to the summary OR and 95 % CI

In the present study, the relationship between the CDH1 -160C/A polymorphism and breast cancer risk was analyzed. The results revealed significant associations between the CDH1 -160C/A polymorphism and breast cancer in the dominant model (CA + AA vs. CC: OR = 1.207, 95 % CI = 1.031–1.412, *P* = 0.019) but not in the other four genotype distributions (A vs. C: OR = 1.231, 95 % CI = 0.992–1.528, *P* = 0.060; AA vs. CC: OR = 1.397, 95 % CI = 0.922–2.116, *P* = 0.115; CA vs. CC: OR = 1.116, 95 % CI = 0.941–1.325, *P* = 0.208; AA vs. CC + CA: OR = 1.338, 95 % CI = 0.850–2.105, *P* = 0.208) (Fig. 2, Table 2).

### Sensitivity analysis

**Fig. 3:**
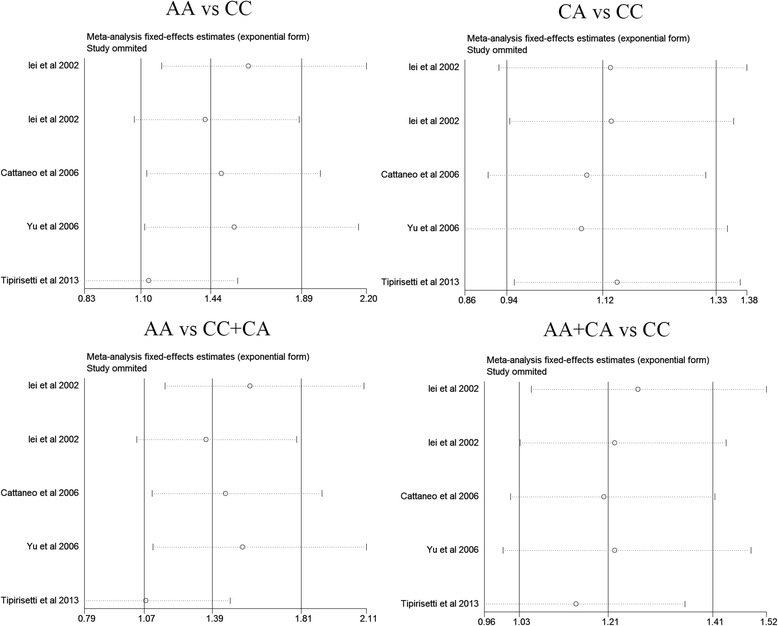
Sensitivity analysis by omitting each study to reflect the influence of each study on pooled OR in different genetic models

To further reinforce our conclusions, a sensitivity analysis was conducted to assess the results’ stability by sequentially omitting each eligible study. The results showed that no other single study influenced the pooled ORs significantly, indicating that our results were statistically robust (detailed data not shown) (Fig. 3).

### Publication bias

The studies’ publication biases were assessed using the Begg and Egger tests. The Egger weighted regression method indicated no evidence of publication bias for all genetic models (A vs. C: *P* = 0.687; AA vs. CC: *P* = 0.680; CA vs. CC: *P* = 0.492; AA + CA vs. CC: *P* = 0.692; AA vs. CC + CA: *P* = 0.698) (Table 3). This result was confirmed using the Begg rank correlation method (A vs. C: *P* = 0.462; AA vs. CC: *P* = 0.806; CA vs. CC: *P* = 0.221; AA + CA vs. CC: *P* = 1.000; AA vs. CC + CA: *P* = 0.806) (Table 3). Hence, there was no obvious publication bias revealed in the current meta-analysis.

**Table 3:** Publication bias test for CDH1 -160C/A polymorphism

Comparisons	Egger test			Begg test
	Coefficient	*P* value	95 % CI	*P* value
A vs C	1.482	0.687	−9.153–12.118	0.462
AA vs CC	−1.157	0.680	−9.239–6.925	0.806
CA vs CC	−0.807	0.492	−4.098	0.221
AA + CA vs CC	0.754	0.692	−4.743–6.250	1.000
AA vs CC + CA	−1.218	0.698	−10.29–7.852	0.806

## Discussion

As a tumor suppressor, E-cadherin expression is frequently reduced or lost in epithelial tumors [3, 5]. This leads to the suboptimal regulation of cell-cell adhesion, loss of cellular polarity, tissue disorganization, tumor progression, and metastasis [3, 4]. Essentially, E-cadherin is the main adhesion molecule of epithelia, its reduced expression being implicated in the epithelial carcinogenic process. Researchers have reported several functional polymorphisms that diminish E-cadherin expression [8–10, 24], among which -160C/A SNP showed transcriptional regulation of the *CDH1* gene in different epithelial cell types.

Cattaneo et al. revealed that -160C/A SNP within the *CDH1* promoter region is a functional polymorphism that affects transcription efficiency in vitro [24]*.* By transient transfection experiments, A allele activity was reduced by 54 and 67 % compared to the -160C allele in cervical and in colon cancer cells, respectively [24]. Therefore, the -160A *CDH1* promoter variant, associated with reduced gene expression, may be regarded as a possible low penetrance susceptibility allele for epithelial tumors. However, studies about the relation between CDH1 -160C/A polymorphism were various. CDH1 -160A allele carriers have a significantly elevated risk for endometrial cancer [24] but not for cervical cancer and ovarian cancer [24, 25]. Thus, we were interested in resolving the controversial question regarding the significance of CDH1 -160C/A in breast cancer pathogenesis. On this basis, the present meta-analysis study focused on breast cancer.

After critically reviewing the five studies on the CDH1 -160C/A polymorphism (1344 cases total, 1569 control), we performed a comprehensive assessment to investigate the significance of CDH1 -160C/A in breast cancer pathogenesis. The results implied a conspicuously significant relationship between the CDH1 -160C/A polymorphism and breast cancer risk under the dominant genetic model (AA + CA vs. CC, OR = 1.207, *P* = 0.019).

These results indicated that the -160 AA genotype increased breast cancer risk, which was consistent with results on other cancer types, such as prostate, urothelial, and bladder cancers [27–29]. However, the results were inconsistent with results on colorectal cancer (CRC) [12, 30], which showed that CDH1 -160C/A polymorphisms could reduce CRC risk. These results revealed that the CDH1 -160C/A polymorphism might have different effects on distinct cancers, and this discrepancy may result from different carcinogenesis mechanisms, such as genetic background, environment exposure, dietary habit, race, or family history.

Concerning breast cancer, Yu et al. found that -160A carriers were 30 % more likely to be breast cancer cases than women with -160C carriers [23]. Conversely, Lei et al. genotyped the -160C/A SNP among 576 cases and 348 controls and found no association with breast cancer risk [17]. Our present study revealed that -160A significantly increased breast cancer risk (CA + AA vs CC, OR = 1.207, *P* = 0.019), which is in good agreement with previous observations on other population samples [17, 31]. However, we enrolled only five studies in the present study. Well-designed, unbiased, and large case-control studies should be performed to acquire a more precise association between the CDH1 -160C/A polymorphism and cancer risk.

As no significant publication bias was revealed in our meta-analysis, sensitivity analysis also revealed that our results were statistically robust. Thus, the results for the association assessed in the present study are relatively convincing since we used a rigorous analytic approach. However, there are still a number of limitations in this meta-analysis.

Firstly, while we identified no publication bias, there is still a possibility that our meta-analysis was biased toward a positive result, since negative results were likely to be unreported. Secondly, our controls were not uniformly defined. Although the healthy population was the main control source, some might be patients with even benign tumors. Thirdly, other factors influencing carcinogenesis should be considered, such as genetic background and environmental and lifestyle factors. Finally, our results had to be interpreted with caution due to unadjusted estimates, so further studies should be conducted to confirm our unadjusted estimates.

## Conclusions

In conclusion, these data provided further evidence that the CDH1 -160C/A polymorphism may represent a risk factor for breast cancer development. Further investigations using a much larger sample, with interactions between the environment and genes taken into account, are still needed to elucidate the findings in this meta-analysis.

## Abbreviations

PCR-RFLP, polymerase chain reaction-restriction fragment length polymorphism; PCR-SSCP, polymerase chain reaction-single-strand conformation polymorphism; SNP, single nucleotide polymorphisms

## Additional file

Additional file 1: The excluded articles and the reasons for exclusion of the articles. (DOC 46 kb)
