# Supplementary material for: The CDH1 -160C/A polymorphism is associated with breast cancer: evidence from a meta-analysis
Source: World J Surg Oncol. 2016 Jun 27;14:169. doi: 10.1186/s12957-016-0927-0 (PMC4924327; doi:10.1186/s12957-016-0927-0)
Supplement: Additional file 1: — The excluded articles and the reasons for exclusion of the articles. (DOC 46 kb) [file 12957_2016_927_MOESM1_ESM.doc]

**Additional file 1**

**The exclude articles and the reasons for exclusion of the articles**

*Reduplicated reports (10 articles)*

1.Tipirisetti Nageswara Rao, Govatati Suresh, Govatati Sowdamani, et al. Association of e-cadherin single-nucleotide polymorphisms with the increased risk of breast cancer: a study in South Indian women.. Genet Test Mol Biomarkers. 2013;17(6):494-500.

2.Lei Haixin, Sjöberg-Margolin Sara, Salahshor Sima, et al. CDH1 mutations are present in both ductal and lobular breast cancer, but promoter allelic variants show no detectable breast cancer risk.. Int J Cancer. 2002;98(2):199-204.

3.Li Yan, Liang Jun, Kang Shan, et al. E-cadherin gene polymorphisms and haplotype associated with the occurrence of epithelial ovarian cancer in Chinese.. Gynecol Oncol. 2008;108(2):409-14.

4.Yu JC, Hsu HM, Chen ST, Hsu GC, Huang CS, Hou MF, Fu YP, Cheng TC, Wu PE, Shen CY (2006) Breast cancer risk associated with genotypic polymorphism of the genes involved in the estrogen-receptor-signaling pathway: a multigenic study on cancer susceptibility. J Biomed Sci 13 (3):419-432

5.Cattaneo F, Venesio T, Molatore S, Russo A, Fiocca R, Frattini M, Scovassi AI, Ottini L, Bertario L, Ranzani GN (2006) Functional analysis and case-control study of -160C/A polymorphism in the E-cadherin gene promoter: association with cancer risk. Anticancer Res 26 (6B):4627-4632

6.Liang Jun, Li Yan, Wang Na[Association of three single nucleotide polymorphisms of the E-cadherin gene with susceptibility to epithelial ovarian carcinoma].. Zhonghua Yi Xue Yi Chuan Xue Za Zhi. 2008;25(2):183-6.

7. Li Yan, Liang Jun, Kang Shan, et al. E-cadherin gene polymorphisms and haplotype associated with the occurrence of epithelial ovarian cancer in Chinese.. Gynecol Oncol. 2008;108(2):409-14.

8. Lei Haixin, Sjöberg-Margolin Sara, Salahshor Sima, et al. CDH1 mutations are present in both ductal and lobular breast cancer, but promoter allelic variants show no detectable breast cancer risk.. Int J Cancer. 2002;98(2):199-204.

9. Zhu Z G, Yu Y Y, Zhang Y, et al. Germline mutational analysis of CDH1 and pathologic features in familial cancer syndrome with diffuse gastric cancer/breast cancer proband in a Chinese family.. Eur J Surg Oncol. 2004;30(5):531-5.

10.Li Yan, Liang Jun, Kang Shan, et al. E-cadherin gene polymorphisms and haplotype associated with the occurrence of epithelial ovarian cancer in Chinese.. Gynecol Oncol. 2008;108(2):409-14.

*Non-English (4 articles)*

1.Nährig J[Practical problems in breast screening. Columnar cell lesions including flat epithelial atypia and lobular neoplasia].. Pathologe. 2008;29 Suppl 2:172-7.

2. Liang Jun, Li Yan, Wang Na[Association of three single nucleotide polymorphisms of the E-cadherin gene with susceptibility to epithelial ovarian carcinoma].. Zhonghua Yi Xue Yi Chuan Xue Za Zhi. 2008;25(2):183-6.

3. Zhou Rong-miao, Wang Na, Sun Dong-lan, et al. [CDH1 polymorphism and its association with the risk of cervical cancer].. Zhonghua Fu Chan Ke Za Zhi. 2009;44(1):56-9.

4. Song Chuan-gui, Huang Chang-ming, Liu Xing, et al. [Association of -160(C-->A) polymorphism in CDH1 gene with gastric cancer risk in Fujian Chinese population].. Zhonghua Yi Xue Yi Chuan Xue Za Zhi. 2005;22(5):557-9.

*Review (6 articles)*

1.Vargas Ana-Cristina, Lakhani Sunil R, Simpson Peter TPleomorphic lobular carcinoma of the breast: molecular pathology and clinical impact.. Future Oncol. 2009;5(2):233-43.

2.Varga Zsuzsanna, Mallon ElizabethHistology and immunophenotype of invasive lobular breast cancer. daily practice and pitfalls.. Breast Dis. 2008;30:15-9.

3. Wang Gui-Ying, Lu Chen-Qi, Zhang Rong-Mei, et al. The E-cadherin gene polymorphism 160C->A and cancer risk: A HuGE review and meta-analysis of 26 case-control studies.. Am J Epidemiol. 2008;167(1):7-14.

4. Wang Lin, Wang Guiying, Lu Chenqi, et al. Contribution of the -160C/A polymorphism in the E-cadherin promoter to cancer risk: a meta-analysis of 47 case-control studies.. PLoS One. 2012;7(7):e40219.

5.Deng Qi-Wen, He Bang-Shun, Pan Yu-Qin, et al. Roles of E-cadherin (CDH1) genetic variations in cancer risk: a meta-analysis.. Asian Pac J Cancer Prev. 2014;15(8):3705-13.

6. Tim Ripperger, Dorothea Gadzicki, Alfons Meindl, Brigitte Schlegelberger. [Breast cancer susceptibility: current knowledge and implications for genetic counselling.](http://www.ncbi.nlm.nih.gov/pubmed/19092773) Eur J Hum Genet. 2009 Jun;17(6):722-31

*Irrelevant to the present study (20 articles)*

1.Wang Hai, Zhou Min, Shi Bizhi, et al. Identification of an exon 4-deletion variant of epidermal growth factor receptor with increased metastasis-promoting capacity.. Neoplasia. 2011;13(5):461-71.

2.Wani Yoji, Saegusa Makoto, Notohara KenjiAberrant nuclear beta-catenin expression in the spindle or corded cells in so-called corded and hyalinized endometrioid carcinomas. Another critical role of the unique morphological feature.. Histol Histopathol. 2009;24(2):149-55.

3.Tomassetti Antonella, De Santis Giuseppina, Castellano Giancarlo, et al. Variant HNF1 modulates epithelial plasticity of normal and transformed ovary cells.. Neoplasia. 2008;10(12):1481-92, 3p following 1492.

4.Nofech-Mozes Sharon, Khalifa Mahmoud A, Ismiil Nadia, et al. Immunophenotyping of serous carcinoma of the female genital tract.. Mod Pathol. 2008;21(9):1147-55.

5.Bullions L C, Notterman D A, Chung L S, et al. Expression of wild-type alpha-catenin protein in cells with a mutant alpha-catenin gene restores both growth regulation and tumor suppressor activities.. Mol Cell Biol. 1997;17(8):4501-8.

6. Risinger J I, Berchuck A, Kohler M F, et al. Mutations of the E-cadherin gene in human gynecologic cancers.. Nat Genet. 1994;7(1):98-102.

Mansor Sorsiah, McCluggage Cervical adenocarcinoma resembling breast lobular carcinoma: a hitherto Undescribed Variant of Primary Cervical Adenocarcinoma.. Int J Gynecol Pathol. 2010;29(6):594-9.

7.Monaco Sara E, Dabbs David J, Kanbour-Shakir AmalPleomorphic lobular carcinoma in pleural fluid: diagnostic pitfall for atypical mesothelial cells.. Diagn Cytopathol. 2008;36(9):657-61.

8.Fadare OluwolePleomorphic lobular carcinoma in situ of the breast composed almost entirely of signet ring cells.. Pathol Int. 2006;56(11):683-7.

9.Kusafuka Kimihde, Ebihara Mitsuru, Ishiki Hiroto, et al. Primary adenoid squamous cell carcinoma of the oral cavity.. Pathol Int. 2006;56(2):78-83.

10. Yoshida Kunihiko, Yoshihara Kosuke, Adachi Sosuke, et al. Possible involvement of the E-cadherin gene in genetic susceptibility to endometriosis.. Hum Reprod. 2012;27(6):1685-9.

11.Aulmann Sebastian, Schnabel Philipp A, Helmchen Birgit, et al. Immunohistochemical and cytogenetic characterization of acantholytic squamous cell carcinoma of the breast.. Virchows Arch. 2005;446(3):305-9.

12. Chien Ming-Hsien, Chou Lin Shih-Shen, Chung Tsung-Te, et al. Effects of E-cadherin (CDH1) gene promoter polymorphisms on the risk and clinicopathologic development of oral cancer.. Head Neck. 2012;34(3):405-11.

13.Sapino A, Frigerio A, Peterse J L, et al. Mammographically detected in situ lobular carcinomas of the breast.. Virchows Arch. 2000;436(5):421-30.

14.Seraj M J, Harding M A, Gildea J J, et al. The relationship of BRMS1 and RhoGDI2 gene expression to metastatic potential in lineage related human bladder cancer cell lines.. Clin Exp Metastasis. 2000;18(6):519-25.

15. Han A C, Soler A P, Knudsen K A, et al. Distinct cadherin profiles in special variant carcinomas and other tumors of the breast.. Hum Pathol. 1999;30(9):1035-9.

16. Chen Bo, Zhou Yong, Yang Ping, et al. CDH1 -160C>A gene polymorphism is an ethnicity-dependent risk factor for gastric cancer.. Cytokine. 2011;55(2):266-73.

17.Pishvaian M J, Feltes C M, Thompson P, et al. Cadherin-11 is expressed in invasive breast cancer cell lines.. Cancer Res. 1999;59(4):947-52.

18.Putz E, Witter K, Offner S, et al. Phenotypic characteristics of cell lines derived from disseminated cancer cells in bone marrow of patients with solid epithelial tumors: establishment of working models for human micrometastases.. Cancer Res. 1999;59(1):241-8.

19. Jonsson Björn-Anders, Bergh Anders, Stattin Pär, et al. Germline mutations in E-cadherin do not explain association of hereditary prostate cancer, gastric cancer and breast cancer.. Int J Cancer. 2002;98(6):838-43.

20. Deman J J, Van Larebeke N A, Bruyneel E A, et al. Removal of sialic acid from the surface of human MCF-7 mammary cancer cells abolishes E-cadherin-dependent cell-cell adhesion in an aggregation assay.. In Vitro Cell Dev Biol Anim. 1995;31(8):633-9.

*Small cases (1 article)*

1.Sarrió David, Moreno-Bueno Gema, Hardisson David, et al. Epigenetic and genetic alterations of APC and CDH1 genes in lobular breast cancer: relationships with abnormal E-cadherin and catenin expression and microsatellite instability.. Int J Cancer. 2003;106(2):208-15.

*No extractable data (3 articles)*

1. Li Yan, Liang Jun, Kang Shan, et al. E-cadherin gene polymorphisms and haplotype associated with the occurrence of epithelial ovarian cancer in Chinese.. Gynecol Oncol. 2008;108(2):409-14.

2. Li L C, Chui R M, Sasaki M, et al. A single nucleotide polymorphism in the E-cadherin gene promoter alters transcriptional activities.. Cancer Res. 2000;60(4):873-6.

3. Blechschmidt Kareen, Kremmer Elisabeth, Hollweck Regina, et al. The E-cadherin repressor snail plays a role in tumor progression of endometrioid adenocarcinomas.. Diagn Mol Pathol. 2007;16(4):222-8.

*Laboratory studies (12 articles)*

1.He Li-Cai, Gao Feng-Hou, Xu Han-Zhang, et al. Ikaros inhibits proliferation and, through upregulation of Slug, increases metastatic ability of ovarian serous adenocarcinoma cells.. Oncol Rep. 2012;28(4):1399-405.

2.Bellyei Szabolcs, Schally Andrew V, Zarandi Marta, et al. GHRH antagonists reduce the invasive and metastatic potential of human cancer cell lines in vitro.. Cancer Lett. 2010;293(1):31-40.

3.Zeineldin Reema, Rosenberg Martina, Ortega Dominic, et al. Mesenchymal transformation in epithelial ovarian tumor cells expressing epidermal growth factor receptor variant III.. Mol Carcinog. 2006;45(11):851-60.

4.Hashimoto M, Niwa O, Nitta Y, et al. Unstable expression of E-cadherin adhesion molecules in metastatic ovarian tumor cells.. Jpn J Cancer Res. 1989;80(5):459-63.

5．Cheng C W, Wu P E, Yu J C, et al. Mechanisms of inactivation of E-cadherin in breast carcinoma: modification of the two-hit hypothesis of tumor suppressor gene.. Oncogene. 2001;20(29):3814-23.

6.Laffin Brian, Wellberg Elizabeth, Kwak Hyeong-Il, et al. Loss of singleminded-2s in the mouse mammary gland induces an epithelial-mesenchymal transition associated with up-regulation of slug and matrix metalloprotease 2.. Mol Cell Biol. 2008;28(6):1936-46.

7.Révillion F, Lhotellier V, Hornez L, et al. Real-time reverse-transcription PCR to quantify a panel of 19 genes in breast cancer: relationships with sentinel lymph node invasion.. Int J Biol Markers. 2008;23(1):10-7.

8.Chua H L, Bhat-Nakshatri P, Clare S E, et al. NF-kappaB represses E-cadherin expression and enhances epithelial to mesenchymal transition of mammary epithelial cells: potential involvement of ZEB-1 and ZEB-2.. Oncogene. 2007;26(5):711-24.

9.Fujiwara Masachika, Horiguchi Miwa, Mori Satoshi, et al. Histiocytoid breast carcinoma: solid variant of invasive lobular carcinoma with decreased expression of both E-cadherin and CD44 epithelial variant.. Pathol Int. 2005;55(6):353-9.

10.Gong Y, Sun X, Huo L, et al. Expression of cell adhesion molecules, CD44s and E-cadherin, and microvessel density in invasive micropapillary carcinoma of the breast.. Histopathology. 2005;46(1):24-30.

11.dit Faute Muriel Affoué, Laurent Luc, Ploton Dominique, et al. Distinctive alterations of invasiveness, drug resistance and cell-cell organization in 3D-cultures of MCF-7, a human breast cancer cell line, and its multidrug resistant variant.. Clin Exp Metastasis. 2002;19(2):161-8.

12.Steelant W F, Recchi M A, Noë V T, et al. Sialylation of E-cadherin does not change the spontaneous or ET-18-OMe-mediated aggregation of MCF-7 human breast cancer cells.. Clin Exp Metastasis. 1999;17(3):245-53.

*Other polymorphism data (4 articles)*

1.Li Yan, Tang Yuanjia, Zhou Rongmiao, et al. Genetic polymorphism in the 3'-untranslated region of the E-cadherin gene is associated with risk of different cancers.. Mol Carcinog. 2011;50(11):857-62.

2. Beeghly-Fadiel Alicia, Lu Wei, Gao Yu-Tang, et al. E-cadherin polymorphisms and breast cancer susceptibility: a report from the Shanghai Breast Cancer Study.. Breast Cancer Res Treat. 2010;121(2):445-52.

3. Becker K F, Reich U, Schott C, et al. Identification of eleven novel tumor-associated E-cadherin mutations. Mutations in brief no. 215. Online.. Hum Mutat. 1999;13(2):171.

4.Kashiwaba M, Tamura G, Suzuki Y, et al. Epithelial-cadherin gene is not mutated in ductal carcinomas of the breast.. Jpn J Cancer Res. 1995;86(11):1054-9.
